# Asthma medication in children who are overweight/obese: justified treatment?

**DOI:** 10.1186/s12887-019-1526-3

**Published:** 2019-05-11

**Authors:** Yvette E. Lentferink, Nienke E. Boogaart, Walter A. F. Balemans, Catherijne A. J. Knibbe, Marja M. J. van der Vorst

**Affiliations:** 1 Department of Pediatrics, St Antonius Hospital, P.O. Box 2500 3430, EM Nieuwegein, The Netherlands; 2 Department of General Practitioners, Leiden University, P.O. 9600, 2300 RC, Leiden, The Netherlands; 3 Department of Clinical Pharmacy, St Antonius Hospital, P.O. Box 2500 3430, EM Nieuwegein, The Netherlands

**Keywords:** Asthma, Obesity, Children, Treatment

## Abstract

**Background:**

The prevalence of asthma and obesity have increased over the last decades. A possible association between these two chronic illnesses has been suggested, since the prevalence of asthmatic symptoms rises with increasing Body Mass Index (BMI). However, asthma is only one of several possible causes of shortness of breath in obese children. The aim of this study is to evaluate the prevalence of overtreatment with asthma medication in a cohort overweight/obese children with respiratory symptoms visiting a pediatric outpatient clinic.

**Methods:**

Children referred to a pediatric outpatient clinic aged ≥4- ≤ 18 years with overweight/obesity (defined as BMI-sds > 1.1) and asthmatic symptoms were included. The diagnosis asthma was evaluated and classified in no, unlikely, probable and confirmed asthma, based on clinical parameters and/or spirometry results. Overtreatment was defined as asthma medication prescribed in participants classified as no or unlikely asthma. And undertreatment as probable or confirmed asthma without asthma medication prescribed .

**Results:**

Three hundred thirty-eight participants were included, of which 92.6% (313/338) had a prescription for asthma medication. Overtreatment was observed in 27.2% (92/338) participants. Nine participants were undertreated.

**Conclusion:**

More than 25% overtreatment with asthma medication was observed in a cohort overweight/obese children with asthmatic symptoms. This finding emphasizes that the diagnosis of asthma must be confirmed before commencement of medication. The diagnosis of asthma should be based on standard questionnaires evaluating asthmatic symptoms, lung functions test and regular reassessments. Further studies concerning overtreatment with asthma medication in normal weight pediatric populations are warranted, to evaluate whether overtreatment is specific for overweight/obese children.

## Background

Obesity and asthma are two major public health problems, affecting children and adults [1–3]. Asthma is diagnosed using a combination of clinical parameters and lung function tests, and treated with β2-agonists and/or Inhaled Corticosteroids (ICS) [4–6]. Spirometry is the most used additional lung function test, which can be reliable obtained from the age of 4–6 year [7].

Since the prevalence of asthma and obesity have increased over the last decades, a possible association between these two chronic illnesses is suggested [1–3, 8, 9]. However, literature is inconsistent on the etiology of this association. It has been suggested that the risk on developing asthmatic symptoms rises with increasing Body mass index (BMI) [1–3]. In addition, some patients demonstrate asthmatic symptoms which are more difficult-to-control with increasing BMI, suggesting a different phenotype [3, 8, 9]. Moreover, the response to asthma medications might be influenced by BMI [3, 9, 10]. Several causes have been postulated to define the higher prevalence of asthma and the altered response to asthma medication in subjects with an increased BMI. Firstly, a reduction in long volume (i.e. restriction), and a reduction/disturbance in chest wall compliance due to fatty infiltration in the thoracic cage, abdomen, and chest wall are suggested [1, 2, 11, 12]. In addition, due to fat excess in the thoracic compartment there is an increased pulmonary blood volume, leading to impairment of the pulmonary function [1]. Furthermore, on account of an excess of adipose tissue, obesity is characterized by a chronic low-grade systemic inflammation, which can result in asthmatic symptoms [1, 12]. Lastly, overdiagnosis of asthma is increasingly suggested as cause for the higher prevalence and differences in asthma medication response, due to an enhanced perception of nonspecific symptoms such as dyspnea [5, 11, 13–18].

Recently high prevalence of overdiagnosis of asthma in children was reported, in primary healthcare centers in the Netherlands [5, 17]. However, little is known about the prevalence of overdiagnosis and consequently overtreatment of asthma in pediatric populations with overweight/obesity in pediatric outpatient clinics. Therefore, the aim of this study is to evaluate the prevalence of overtreatment with asthma medication in a cohort overweight/obese children with asthmatic symptoms visiting a pediatric outpatient clinic.

## Methods

### Study design and subjects

This retrospective cross-sectional observational study was approved by the Medical Ethical Committee of the St. Antonius Hospital, Nieuwegein/Utrecht, the Netherlands (W17.013), and performed in accordance with the Helsinki Declaration of 1975, as revised in 2008. Need for written informed consent was formally waived by the ethics committee as only data obtained from routine clinical care were used and analyzed anonymously (W17.013). Patients who visited the pediatric outpatient clinic of the St. Antonius Hospital, a non-academic teaching hospital, referral centre for primary care physicians, centrally located in the Netherlands, between January 2013 and July 2016 were screened for the Diagnosis treatment combination code (DBC code) “adipositas” and/or “asthma”. Patients aged ≥4- ≤ 18 years, with overweight/obesity, and ‘asthmatic symptoms’, were included. A patient was considered as having asthmatic symptoms if β2-agonist and/or ICS were prescribed and/or diagnosis asthma was recorded in the medical file. Patients with weight affecting disorders, chest wall abnormalities and incomplete data were excluded. All potential participants were included once, even if the potential participant had multiple DBC codes due to multiple visits during the inclusion period.

### Measurements

From electronic medical records demographics and anthropometric measurements (i.e. date of birth, sex, date of intake or date of spirometry, prescribed asthma medication, height, and weight) were retrieved. In addition, information on clinical asthma symptoms and results of spirometry were gathered. Anthropometric measurements had to be available from date of intake at the pediatric outpatient clinic and/or within a range of two months from the date of spirometry. Weight and height were measured using a digital scale (Seca, Hamburg, Germany) with an accuracy of 0.05 kg and a digital stadiometer with a precision of 0.1 cm (DGI 250D, De Grood, Nijmegen, the Netherlands), respectively. BMI and corresponding age and sex adjusted BMI standard deviation score (BMI-sds), and height-sds were calculated using the TNO growth calculator for professionals (https://groeiweb.pgdata.nl/calculator.asp). Patients were classified as having overweight or obesity, according to the national cut-off values for BMI-sds, defined as BMI-sds > 1.1 and ≤ 2.3, and BMI-sds > 2.3, respectively [19]. The national cut-off values are corresponding with the cut-off values defined by the International Task Force Obesity (IOTF) (= the World Obesity Federation) and with the cut-off values defined by the Center of Disease Control and Prevention (CDC) [20, 21]. The IOTF uses specific pediatric BMI equivalent of the adult cut-off values for overweight (BMI > 25) and obesity (BMI > 30) and the CDC uses the >85th (overweight) and > 95th (obesity) percentile both for age and sex.

The diagnosis asthma was evaluated, based on data extracted out of medical files, by categorizing participants into 4 groups (i.e. confirmed asthma, probable asthma, unlikely asthma, no asthma), according to the definition of Looijmans et al. [5]. The diagnosis asthma was considered as confirmed if participants ≥6 years of age had recurrent dyspnea or wheezing, with reversible bronchial obstruction confirmed by a pediatric specialist in clinical outpatient setting and/or with spirometry. In participants < 6 years of age, who could not perform a spirometry, the diagnosis asthma was considered as confirmed if they had recurrent dyspnea of wheezing, with reversible bronchial obstruction confirmed by a pediatric specialist in clinical outpatient setting. Participants were considered as probable asthma if they had a suggestive medical history and physical examination during an exacerbation, but spirometry was not performed or without significant reversibility. Or if participants had a suggestive medical history and asthma medication was prescribed chronically but no recent history of asthma exacerbations (i.e. properly regulated asthma). Asthma was considered unlikely if participants had no asthma exacerbation, no prescription of ICS, and used no or very little short acting β2-agonists with doubtful effect on asthmatic symptoms such as dyspnea or wheezing. No asthma was considered if the diagnosis was ruled out by a pediatric specialist based on medical history, physical examination, and/or lung function tests.

Overdiagnosis and consequently overtreatment was defined as asthma medication prescribed (reliever and or ICS) in participants classified as unlikely asthma or no asthma [5]. Underdiagnosis and consequently undertreatment was defined as no prescribed asthma medication in participants classified as probable or confirmed asthma [5]. A diagnosis asthma based on clinical symptoms and/or suggestive medical history will be referred to as clinical diagnosis.

All spirometries in the St. Antonius Hospital, conducted from the age of 4 years onwards, were performed according to the European Respiratory Society (ERS) guidelines under supervision of a specialized laboratory assistant. Only technically well performed spirometries were evaluated. The first performed spirometry was used, however, when technically inadequate, results of a subsequent spirometry were obtained. Spirometries with uncertain reliability were blinded, and thereafter assessed for verification by a pediatric pulmonologist. In case of short acting β2-agonists use as standard treatment, medication was withheld for at least 8 h before the test and long acting β2-agonists for at least for 24 h, respectively. According to the standard procedure spirometry of the St. Antonius Hospital, maintenance therapy with ICS was allowed to be continued. Values obtained from spirometries were: Forced Vital Capacity (FVC) and Forced Expiratory Volume in one second (FEV1), both recorded before and after medication administration (β2-agonist) in order to assess reversibility. Values were recorded in litres and converted to percent of predicted values and Z-scores, adjusted for age, length, gender and ethnicity, using the Excel Individual Calculator of the Global Lungs Initiative 2012 (GLI-2012) [22]. Since ethnicity was not standard documented, all participants were categorized as Caucasian. For accurate interpretation of the spirometry values age and height were noted with a precision of one decimal, since 1% discrepancy in height led to deviations in FEV1 and FVC of 2.1–2.4% in the GLI-2012 [23, 24]. Reversible airflow obstruction, and thereby a positive spirometry, was defined as an increase in percentage of predicted FEV1 (adjusted for age, length, gender, ethnicity) of ≥12% after administration of 400 mcg of salbutamol [4–6, 25].

### Statistical analysis

Statistical analysis was performed using IBM SPSS Statistics, version 24 (IBM SPSS Statistics, Chicago, IL, USA). Normally distributed continuous parameters were reported as mean ± standard deviation and nonparametric continuous parameters as median with range. Categorical data were expressed as frequencies with percentage. To compare baseline characteristics between the 4 asthma subgroups the Kruskal Wallis test was used to compare continuous variables, and the Chi-squared test for categorical data. A α-level of 5% was considered significant for all statistical tests.

## Results

**Fig. 1:**
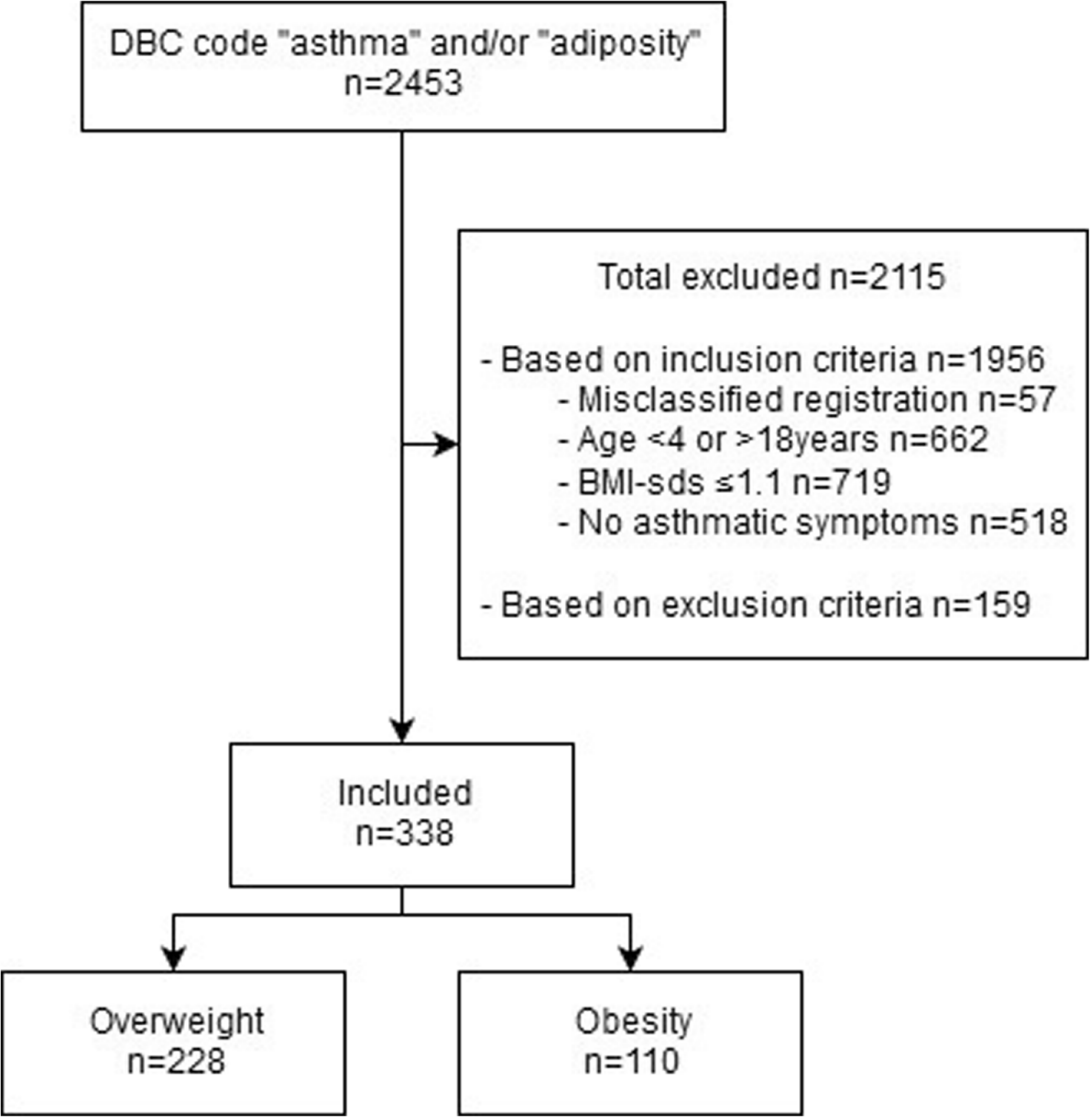
Flowchart of the study population

Figure 1 shows that in total 2453 DBC codes “adiposity” and/or “asthma” were identified and considered for inclusion. For various reasons, 2115 patients were excluded. Of the 338 patients included, 67.5% (228/338) had overweight and 32.5% (110/338) obesity.

Table 1 shows the baseline characteristics of the total population with a median age of 8.9 years, and a BMI-sds of 1.99. Asthma medication (short acting β2-agonists and/or ICS) was prescribed in 92.6% (313/338) of the participants, and spirometry was performed in 74.3% (251/338).

**Table 1:** Baseline characteristics of the total population *n* = 338

Age (years)	8.9 (5.4)
Male n (%)	209 (61.8)
Height (cm)	136.9 (99.5–196)
Height-sds	0.02 (−2.95–2.65)
Weight (kg)	39.3 (15.7–127.0)
BMI (kg/m^2^)	20.63 (16.65–39.81)
BMI-sds	1.99 (1.11–5.24)
BMI-categories	
- Overweight	228 (67.5)
- Obesity	110 (32.5)
Spirometry performed	251 (74.3)
Prescribed asthma medication	313 (92.6)
- β2-agonists	100 (29.6)
- ICS	38 (11.2)
- Both	175 (51.8)
- No	25 (7.4)

Data presented as number (%) or median with range, age presented as median with interquartile range. *Sds* standard deviation score*, BMI* body mass index*, ICS* Inhaled Corticosteroids

Table 2 shows the characteristics of participants according to the categorization of asthma diagnosis. No significant differences were observed between the four subgroups in age, sex, height, weight, BMI-categories, or amount of performed spirometries. By definition, significant differences were observed in clinical diagnosis, positive spirometries, and medication prescribed.

**Table 2:** Baseline characteristics according to categorization of asthma diagnosis *n* = 338

	No *n* = 64 (18.9)	Unlikely *n* = 44 (13.1)	Probable *n* = 168 (49.7)	Confirmed *n* = 62 (18.3)	*P*-value
Age (years)	8.8 (4.2–16.2)	8.5 (4.1–17.1)	9.5 (4.2–16.9)	7.8 (4.0–17.7)	0.119
Male n (%)	34 (53.1)	27 (61.4)	108 (64.3)	40 (64.5)	0.444
Height (cm)	135.5 (103.3–178.5)	135.2 (104.8–177.1)	139.0 (105.0–196.6)	132.2 (99.5–178.6)	0.226
Height-sds	0.03 (−2.95–1.82)	0.24 (−1.75–2.57)	−0.09 (− 2.37–2.65)	0.10 (− 2.23–1.51)	0.433
Weight (kg)	40.9 (19.2–80.6)	35.8 (18.6–85.4)	40.2 (19.1–103.0)	35.9 (15.7–127.0)	0.212
BMI (kg/m^2^)	21.06 (16.72–30.22)	20.36 (16.65–30.17)	20.84 (16.65–36.35)	19.89 (17.76–39.81)	0.369
BMI-sds	2.09 (1.11–4.23)	1.97 (1.13–4.36)	1.96 (1.11–5.24)	1.93 (1.11–4.19)	0.663
BMI-categories					0.905
- Overweight	41 (64.1)	29 (65.9)	115 (68.5)	43 (69.4)	
- Obesity	23 (35.9)	15 (34.1)	53 (31.5)	19 (30.6)	
Spirometry	43 (67.2)	28 (63.6)	131 (78.0)	49 (79.0)	0.101
Reversibility ≥12%	–	–	–	40 (64.5)	< 0.001
Clinical diagnosis	–	–	168 (100)	56 (90.3)	< 0.001
Clinical diagnosis and reversibility ≥12%	–	–	–	34 (54.8)	< 0.001
Prescribed asthma medication n (%)					< 0.001
- β2-agonist	25 (39.1)	37 (84.1)	24 (14.3)	13 (21.0)	
- ICS	4 (6.3)	–	20 (11.9)	14 (22.6)	
- Both	26 (40.6)	–	116 (69.0)	34 (54.8)	
- No	9 (14.1)	7 (15.9)	8 (4.8)	1 (1.6)	

Data presented as number (%) or median with range*. BMI* body mass index*, sds* standard deviation score*, ICS* Inhaled Corticosteroids

In total 32% (108/338) of the participants were classified as no asthma or unlikely asthma. In this group asthma medication was prescribed in 85.2% (92/108) of the participants. ICS was prescribed 32.6% (30/92). According to the used definition 27.2% (92/338) overtreatment with asthma medication was observed in this cohort. Of the 68.0% (230/338) participants classified as probable or confirmed asthma, asthma medication was prescribed in 96.1% (221/230). However, in 3.9% (9/230) of the participants with probable or confirmed asthma no asthma medication was prescribed and were therefore by definition undertreated. Participants with probable or confirmed asthma had in most cases a clinical diagnosed asthma (97.4%; 224/230). Spirometry confirmed asthma was observed in 17.4 (40/230). Six (15%) participants with positive spirometry did not have a clinical diagnosis.

No significant differences were observed in overtreatment prevalence between children with overweight and obesity (26.3% vs. 29.1%, *p* = 0.793).

## Discussion

The prevalence of asthmatic symptoms and consequently the use of asthma medication rises with increasing BMI [1–3, 8, 9]. Several causes for this higher prevalence of asthma in patients with obesity have been postulated, such as lower vital capacity and restrictive lung function patterns, chronic low-grade systemic inflammation, and overdiagnosis [5, 11, 13–18]. The aim of this study was to evaluate the prevalence of overtreatment with asthma medication in overweight/obese children at a pediatric outpatient clinic.

In this study in more than 25% of the children with overweight/obesity asthma medication was prescribed without a confirmed or probable diagnosis of asthma. Overtreatment is consequently the result of overdiagnosis. In literature high prevalence of overdiagnosis and thereby overtreatment has been reported in both non-obese and obese adults, and children [5, 13, 17]. Two recent studies performed in primary healthcare centers in the Netherlands, reassessed whether the asthma diagnosis in children was given correctly and whether pharmacological asthma therapy was indicated [5, 17]. Both studies evaluated the validity of registered asthma diagnosis based on information on asthma-related characteristics reported in medical files such as (episodic) wheezing, dyspnea, cough, family history for atopic diseases, sensitivity for non-specific irritants, allergy test, and reversibility in pulmonary function test [5, 17]. Based on an algorithm of combination of symptoms, Pauwelse classified patients into probable asthma, unlikely asthma, and insufficient data for the diagnose asthma [5, 17]. Looijmans classified patients as confirmed, probable, unlikely, and no asthma [5, 17]. In addition, it was documented which asthma characteristics were most frequently reported to support the diagnosis asthma [5, 17]. They showed a prevalence of overdiagnosis and thereby overtreatment up to 53.2% [5, 17]. Erroneous asthma diagnosis occurred significantly more often in children before the age of 6 years, in which the diagnosis was in most cases exclusively based on one or two episodes of wheezing and/or dyspnea [17]. This underlines the need for regularly reassessment of the diagnosis in children, especially since it is suggested that asthmatic symptoms may decrease with increasing age [13]. In a community based study in children from Toronto Canada a high prevalence of asthma overdiagnosis was observed as well [26]. Only 53% of the children with a clinical diagnosis asthma fulfilled the criteria to confirm the diagnosis asthma (i.e affirmative clinical diagnosis by asthma expert physician and observed reversible airway obstruction) [26]. Since this number is in concordance with the results in primary care health centers from the Netherlands, we assume that the prevalence of asthma diagnosis in children in other countries will be comparable. Also in adults, high prevalence of overdiagnosis has been observed in individuals with and without obesity (31.8 vs. 28.7%) [13]. The suggestion that the association between asthma and obesity is caused by enhanced perception of “asthmatic” symptoms could not be confirmed in that study, since no difference between obese and non-obese individuals was observed [13]. We did not observe any differences in prevalence of overtreatment between children with overweight and obesity, possibly because differences in BMI-sds were too small. However, since we only included patients with overweight/obesity no pronunciations can be made whether there are differences in overtreatment between children with overweight/obesity in comparison with children with normal weight. The prevalence of overtreatment observed in the current study corresponds with the adult study [13], but is lower than that of the studies performed in the general practice [5, 17], which might be caused by differences in study design and population. Our population was recruited from a pediatric outpatient clinic, a referral center for patients from primary health care centers with more severe or therapy resistant asthma. This explains the high prevalence (> 90%) of asthma medication prescribed and thereby less undertreatment. In addition, we evaluated the diagnosis of asthma after the first visit at the pediatric outpatient clinic, in contrast with the studies performed in the primary healthcare centers, and our population was younger (8.9 vs. 10.7 years). Hereby a possible decrease of asthmatic symptoms over the years could not be take into account, causing possibly a relative underestimation of overtreatment in our population. In this study the diagnosis of asthma was not re-evaluated. Re-evaluation of patients enrolled at 4–6 years of age might have been of added value, since the diagnosis at this age might be influenced by subjective assessment by the attending physician and may have led to bias. Since the diagnosis asthma was only evaluated after the first visit at the pediatric outpatient clinic, the final diagnosis of patients classified as unlikely or no asthma were not studied. However, several theories why overtreatment may be more prevalent in populations with overweight/obesity are postulated, which could also apply to our population. Subjects with overweight/obesity might report asthma-like symptoms, which could be caused by a poor physical condition, or due to effort limitation caused by the overweight/obesity itself [13, 18]. In addition, reduced chest wall compliance, due to fat infiltration, results in reduced lung volumes and increased work of breathing and increased energy and oxygen cost of breathing [13]. This could all mimic true asthmatic symptoms, whereby a clinical diagnosis asthma is made more easily.

There are obvious consequences associated with overdiagnosing asthma. This includes the lost opportunity to investigate and/or treat the true cause of respiratory symptoms properly, potential exposure to adverse effects of asthma medications [27], the cost of asthma medications, and the social consequences and psychological impact being labeled with a chronic respiratory disease [13]. In children the diagnosis is mainly based on clinical parameters and treatment is frequently started on an empirical basis, although additional lung function tests are recommended [4, 27, 28]. This was also observed in the current study, since 97.4% of the participants diagnosis asthma was based on clinical parameters, and only 17.4% confirmed by spirometry. A recent study showed that in adults with morbid obesity the use of additional lung function test is necessary to confirm or to exclude the diagnosis asthma, due to prevalence of asthma-like symptoms [18]. The use of additional lung function test in children with overweight/obesity seems therefore useful to give an accurate diagnosis and to prevent overdiagnosis and thereby overtreatment with asthma medication. Moreover, regular reassessment of the diagnosis in children seems warranted since asthma can change over time and be outgrown. On the other hand, undertreatment could even be more harmful, since it might increase the risk on asthma exacerbations, decrease quality of life and limit children’s exercise capacity due to asthma symptoms during exercise and sports. In our population nine participants had a probable or confirmed asthma, but no prescription for asthma medication. Since it is known that asthma also may interfere with exercise, it is of great importance to optimize asthma treatment in those with overweight/obesity and true asthma.

The FEV1/FVC ratio (Tiffaneau index), commonly used in adults to diagnose asthma, changes with age and is therefore a less reliable value in children [6, 24, 29]. In young children this ratio can be as high as 0.96, so use of the commonly used fixed ratio of 70% will substantially underestimate airflow limitation [4, 6, 24, 29]. To confirm the diagnosis asthma through spirometry, we therefore defined reversible airflow obstruction as an increase of percentage predicted FEV1 of ≥12% [4–6, 25]. However, a substantial part of children with asthma, do not meet the criteria of ≥12% reversibility, nor have signs of airflow limitation [4]. Therefore, asthma guidelines advise to use both clinical symptoms and additional lung function tests to ensure an accurate diagnosis, taking the variable expression of asthmatic symptoms into account [4, 6, 25]. However, in patients with obesity an enhanced perception of nonspecific symptoms such as dyspnea and decreased exercise performance are reported, which may easily lead to overdiagnosis and subsequent overtreatment, especially when clinical symptoms predominate as the basis for the diagnosis [11, 13–16].

### Limitations

In this single center study we evaluated whether asthma treatment was preceded by an established asthma diagnosis based on international guidelines. We focused on children at a pediatric outpatient clinic with obesity/overweight and asthmatic symptoms, a population which was not previously evaluated according to the authors’ knowledge. However, certain limitations due to the retrospective study design must be considered. Some potentially eligible individuals could not be included in the study, which have led to a reduction in population size, because their asthma was regulated in primary healthcare centers. Therefore important characteristics such as height, weight, medication use, and spirometry values at time the diagnosis asthma was considered, were often incomplete or missing. Moreover, due to the absence of a standard questionnaire to evaluate asthmatic parameters during the intake at the outpatient clinic, the diagnosis of asthma of the participants could be based on different combinations of parameters and be influenced by subjective assessment by the attending physician which is especially of importance in children under the age of 6 years. Consequently, some children were diagnosed with asthma on less parameters than others, leading to under or overestimation of overtreatment. On the other hand, overdiagnosis could also be overestimated since asthmatic symptoms in medical records are not always described in detail. In addition, not all participants underwent a spirometry as standard care, and additional lung function test such as the histamine or methacholine challenge test could not be taken into account since this was only conducted in only a few participants. Spirometry results were converted into age, height and sex adjusted percent of predicted values using the GLI-2012 [22], however, no clear correction could be made for ethnicity since this was not standard recorded in medical files. All participants were classified as Caucasian, since this is the predominant ethnicity of patients visiting our pediatric outpatient clinic. This might have influenced the results, however most variability in GLI is observed in African Americans and South East Asians [30], who are hardly seen at our pediatric outpatient clinic. Furthermore, underlying pulmonary disease, such as chronic inflammation, bronchopulmonary dysplasia due to par example premature birth were not taken into account. Lastly, several other causes such as restrictive lung disease and chronic low-grade systemic inflammation have been postulated to explain the higher prevalence of asthmatic symptoms in overweight/obese patients, leading to overdiagnosis of asthma. However, in this retrospective cross-sectional observational study these causes were not evaluated in the 92 patients, who were classified as unlikely or no asthma.

## Conclusion

More than 25% overtreatment with asthma medication was observed in a cohort overweight/obese children with asthmatic symptoms. This emphasizes that the diagnosis of asthma must be confirmed before commencement of medication. The diagnosis of asthma should be based on standard questionnaires evaluating asthmatic symptoms, lung functions test and regular reassessments. Further studies concerning overtreatment with asthma medication in normal weight pediatric populations are warranted, to evaluate whether overtreatment is specific for overweight/obese children.

## Abbreviations

BMI: Body Mass Index
DBC: Diagnosis treatment combination
FEV1: Forced Expiratory Volume in one second
FVC: Forced Vital Capacity
GLI: Global lung initiative
ICS: Inhaled Corticosteroids
sds: Standard deviation score
